# Primary and revision lumbar discectomy: A three-year review from one center

**DOI:** 10.4103/0019-5413.40254

**Published:** 2008

**Authors:** KN Acharya, TS Senthil Nathan, J Renjit Kumar, K Venugopal Menon

**Affiliations:** “Dwaraka Institute of Spine Care”, BMA/Moka Road, Gandhinagar, Bellary - 583 103, Karnataka, India; 1 Department of Orthopedics, Amrita Institute of Medical Sciences, Cochin - 682 026, Kerala, India

**Keywords:** Lumbar discectomy, herniated disc, revision discectomy

## Abstract

**Background:**

Despite variations in technique, the results of primary and revision lumbar discectomy have been good. The aim of this study was to retrospectively review cases of primary and revision lumbar discectomy performed in our institute over a three-year period.

**Materials and Methods:**

The case records of 273 patients who underwent lumbar discectomy between January 2001-2004 and fulfilled our inclusion and exclusion criteria were reviewed. Of these, 259 were primary discectomies and 14 were revision surgeries. Recurrence was defined as ipsilateral disc herniation at the previously operated level. Demographic parameters, magnetic resonance imaging of the disc, patient satisfaction and rate of recurrence were analyzed.

**Results:**

The primary surgery group had 52 (20.08%) contained and 207 (79.92%) extruded or sequestered discs, while the numbers in the revision group were three (21.43%) and 11 (78.57%) respectively. “Satisfactory” outcome was noted in 96.5% of the primary surgeries, with a recurrence rate of 3.5%. In the revision group 78.6% had “satisfactory” outcome. In 9.4% of the primary group we encountered complications, while it was 21.43% in the revision group.

**Conclusions:**

Lumbar discectomy is a safe, simple and effective procedure with satisfactory outcome in 96.5% of primary disc surgery and 78.6% of revision disc surgery.

## INTRODUCTION

The surgical treatment of symptomatic lumbar disc herniation is now focused on nerve root decompression with preservation of the bony and ligamentous stabilizers of the spine. Various techniques of discectomy have been devised in accordance with this principle and irrespective of the technique used, primary disc surgeries have generally known to give good results.^1^–^4^ The rate of recurrent disc herniation ranges from 3-20%^2^,^5^,^6^ and it constitutes a major cause of failed back syndrome. Satisfactory results with revision disc surgery vary from 50-90%^5^–^7^ and there is no consensus on which technique gives the best results. In this study, we report the retrospective review of the cases of primary and revision lumbar discectomy performed in our institute over a period of three years.

## MATERIALS AND METHODS

We retrospectively reviewed all patients who underwent lumbar discectomy in our institute between January 2001-2004. All cases were operated by senior author (KVM). The inclusion criteria were: (a) intractable leg pain associated with positive root stitch signs and not responding to conservative treatment for a minimum of 12 weeks; (b) signs of nerve entrapment and correlating magnetic resonance imaging (MRI) scans; (c) minimum follow-up of one year. Recurrence was defined as a disc herniation on the same side, at a previously operated level. Patients presenting with cauda equina syndrome, only back pain without leg pain and those who had undergone previous additional procedures (like fusion etc) and those with multiple level disc surgery were excluded from the study.

### Operative procedure

The surgical steps were common for all the cases and were as follows. With the patient in the knee chest position under general anesthesia, the level of the disc was marked with X-ray guidance. Through a one-inch, straight, longitudinal midline incision the paraspinal muscles were elevated to approach the inter-laminar space on the affected side. A right-angled Hohman's retractor (acetabular type) was used and held in place with a Charnley weight (1 kg) and chain device. A unilateral flavectomy exposed the nerve root which was retracted medially or laterally depending on the position of the disc and through a transverse annulotomy all the loose disc material was removed. The midline ligaments, facets and laminae were left undisturbed. The operating microscope was not used in any of the cases.

Revision surgery differed in that the exposure of the spinal canal commences from the medial border of the inferior facet rather than the midline ligament and a partial facetectomy (< 25%) was performed to expose the lateral part of the annulus. In all cases the annulus was then incised laterally, without retracting the fibrous scar on its medial aspect which contained the nerve root. Our technique of lumbar discectomy had a few variations from the standard technique described by McCulloch.^7^ The knee-chest position used by us (as against the kneeling position) offers the advantage of opening up the interlaminar space; the lumbo-dorsal fascial incision was linear and immediately adjacent to the midline (as against the curved incision); and despite the small size of the incision we did not use an operating microscope. The use of a bent Hohman's retractor with the weight and chain device provided adequate exposure of the lamina above and below without interference on the medial side (as in the self-retaining Meyerding retractor etc.). The technique also had the advantage that it did not entail a long learning curve, unlike that of endoscopic discectomy.

The patients are mobilized out of bed on the same evening and discharged home on the third postoperative day (range two to seven days). All patients were reviewed on Day 14 for suture removal and initiation of spinal stabilization exercises and then at six weeks, 12 weeks, six months and one year.

Parameters such as gender, age, level and side of discectomy and clinical outcomes were entered into a database for analysis together with diagnostic parameters. The appearance of the disc on the MRI was categorized as “contained” (focal extension of the posterior margin of the disc beyond the adjacent vertebral bodies), “extruded” (presence of disc fragment migrated through a defect of the posterior longitudinal ligament, but still connected to the disc) or “sequestered” (herniated tissue was no longer connected to the disc). The rate of recurrence was also reviewed. The clinical outcome at the end of the first year after surgery was assessed according to McCulloch's “functional grades”.^7^ Grade I (complete relief of symptoms) and Grade II (mild discomfort; able to participate in all activities) were grouped as “satisfactory”; while Grade III (better than preoperative status, significant limitation of activities and/or requiring medications and/or bracing) and Grade IV (no better than preoperative status, unable to return to work) as “unsatisfactory”. Clinical outcome measurement instruments based on scoring could not be used as this was a retrospective study based on hospital records.

## RESULTS

Of the 315 lumbar discectomies performed during the study period, 273 (86.7%) patients fulfilled the inclusion criteria and were included in the analysis. Of these, 259 patients had primary discectomy, while the remaining 14 were revision surgeries. The follow-up varied from 1-4.5 years, but the clinical outcomes were evaluated at the end of one year from surgery in all the patients.

In the primary surgery group there were 197 (76.1%) men and 62 (23.9%) women, while the revision group had 12 (85.1%) men and two (14.3%) women. The age distribution was similar in both the groups, with the maximum numbers (44% and 42.9%) in the fourth decade of life. Of the 14 patients who underwent revision surgery, eight belonged to the primary surgery group of this study, while the remaining six had undergone their primary surgery elsewhere. Excluding the two residual discs, the remaining 12 patients with recurrences gave a history of complete resolution of symptoms after the primary surgery. Amongst these, eight (66.7%) had a history of a precipitating event prior to onset of pain, seven (58.3%) a history of regular alcohol consumption, nine (75%) a history of regular smoking and all 12 had a definitive history of significant lack of physical activity. The time interval between the primary and revision surgery ranged from three months to 10 years (average 2.6 years).

**Figure 1:**
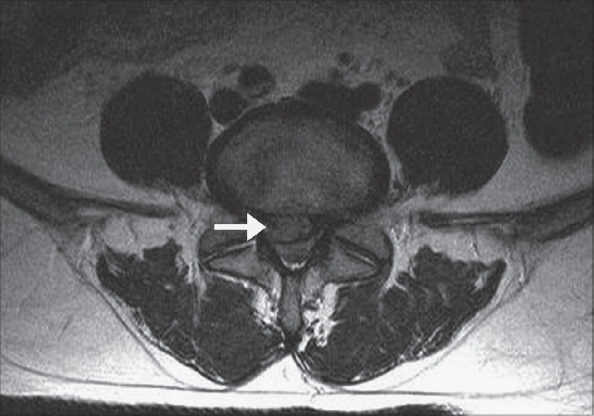
Axial MR image of a patient in the primary discectomy category - MRI showing extruded disc (arrow)

**Figure 2:**
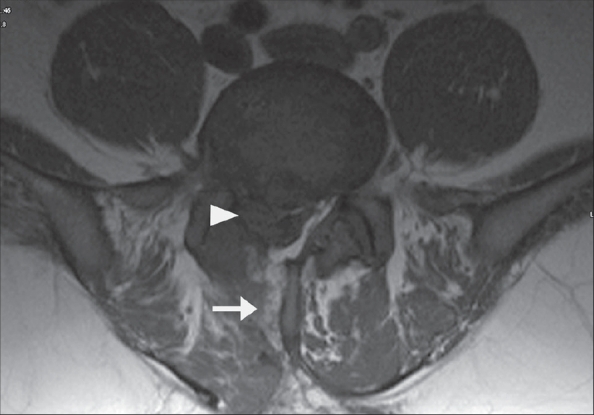
Axial MR image of a patient in the revision category - MRI showing track of previous procedure (arrow) and sequestered fragment in canal (arrow head)

Primary surgery involved the L4-5 disc in 183 (70.7%), L5-S1 disc in 65 (25%) and L3-4 disc in 11 (4.2%). Similarly, of the 14 revision surgeries 11 (78.6%) were at the L4-5 level, two (14.2%) at L5-S1 and one (7.1%) at L3-4. The primary surgery group had 52 (20.1%) contained discs, 89 (34.4%) extruded discs [Figure 1] and 118 (45.6%) sequestered discs. In the revision surgery group there were three (21.43%) contained discs, eight (57.1%) extruded discs and three (21.4%) sequestered discs [Figure 2]. One of the extruded discs appeared as a hard calcified mass intraoperatively, but histopathological examination confirmed it to be inter-vertebral disc tissue. In three revision cases, there was a membrane similar to the previously resected ligamentum flavum, which had to be re-excised.

Of the 259 patients who underwent primary lumbar discectomy in our center, 250 (96.5%) had “satisfactory” outcomes, while nine (3.4%) had “unsatisfactory” results. All patients with “unsatisfactory” outcomes were re-evaluated with an MRI and eight of them underwent revision surgery in due course of time and the remaining one was lost to follow-up. In the revision surgery group, 11 (78.5%) patients had complete relief of symptoms, while three patients (21.4%) had an “unsatisfactory” outcome. Over the course of time one of them had exacerbation of symptoms and underwent a second revision surgery.

There were 25 complications amongst the 259 primary discectomies, giving us a complication rate of 9.65%. These included eight (3.1%) instances of inadvertent dural puncture, none requiring a repair and managed with fat graft and bed rest; three (1.2%) cases of nerve root injury (visualized intraoperatively), without motor deficit and all recovering in the postoperative period; and a 1.93% incidence of postoperative infection - four superficial wound infections and one deep infection, all managed with antibiotics only. Intraoperative epidural bleeding, significant enough to hinder surgery and requiring the use of bipolar diathermy, was encountered in nine (3.5%) patients. During the 14 revision surgeries we encountered three (21.4%) complications. These included dural tear in two cases (not repaired) and wrong level exploration in one case, which was identified and rectified intraoperatively. There were no instances of nerve root avulsion or postoperative infection in the revision group.

## DISCUSSION

The incidence of recurrent disc herniation has been reported to vary from 3-20%^2^,^5^,^6^,^8^ depending upon what constitutes a recurrence. Ipsilateral herniations at a previously operated level are the commonest type of recurrences (60-8%)^9^ and we have included only these in the revision group because we would like to highlight the utility of the same technique of discectomy for both primary surgery and revision surgery through the scarred tissue. The inclusion of contralateral herniations and recurrence at a different level would mean that the surgery was done through virgin tissue. The incidence of ipsilateral recurrences amongst the primary lumbar discectomies done in our institute was nine out of 259 (3.5%) and this is comparable with that described in the literature though the numbers may be skewed in our favor because of the short follow-up.

Risk factors like young age, male gender, smoking and traumatic events have been described for recurrent disc herniations.^6^ Cinotti *et al.*,^5^ reported that men with markedly degenerated discs and those with an isolated injury or a precipitating event were more predisposed to ipsilateral recurrences but not contralateral recurrent herniations.^10^ In our series, there was a large number of patients (66. 7%) who had a definite precipitating event for the recurrence. Cinotti *et al.*^5^ and Suk *et al.*,^6^ have hypothesized that the annular incision of the primary surgery makes the operated disc more susceptible, especially under conditions of mechanical loading and this is probably why recurrence is more common in younger men. Seventy-five per cent of the revision group in our series also had a history of regular smoking. To what extent the lack of physical activity (exercises) contributes to the recurrence of disc herniation is not known, but an interesting observation in our study was that none of the patients in the revision group had any form of regular exercise and all of them led a sedentary lifestyle.

The natural history of lumbar disc herniation may play a role in the type of disc encountered in surgical series. Our retrospective analysis of 273 surgical cases had a large majority of extruded and sequestered discs -80% in the primary surgery group and 78.6% in the revision group. Given the facts that most disc herniations resorb over time, that larger and uncontained (extruded and sequestered) herniations tend to regress to a greater extent^11^–^13^ and that ours is a tertiary referral hospital (most patients do not come to us at the onset of symptoms), we derive that three months of non-operative treatment is probably not adequate for resolution of symptoms, even in extruded and sequestered discs. This is contradictory to the recommendations in the literature.^7^,^12^ There are two possible reasons for the greater number of extruded and sequestered discs in our series; they may have had more aggressive symptoms necessitating surgical treatment and perhaps we have a bias to prolonging non-operative treatment for contained discs.

Clinical outcomes in primary disc surgeries have generally known to be good but the same is not the case with revision disc surgeries.^2^,^10^,^14^ In the current series, 78.6% of the revision group had “satisfactory” outcomes, which is comparable with results described in the literature.^5^,^6^,^14^ This justifies our philosophy of using the same lumbar discectomy procedure in the management of revision disc surgeries rather than a more extensive procedure.

The complication rates with our technique of lumbar discectomy are comparable with those described in the literature,^2^,^15^ though the definitions are variable. Significant epidural bleeding has been considered as a complication because it prolongs the surgical time (which is a limiting factor with the knee-chest position) and it is our belief that the use of bipolar diathermy increases scarring. Our complications during revision surgery are higher (21.4%) than those encountered during primary surgery (9.65%) and though this is corroborated in the literature,^2^,^8^,^14^ this could be due to the small number of patients in the revision group. The incidence of accidental durotomy was 3.1% in the primary group and 14.3% in the revision surgery group and was similar to that described by Morgan-Hough *et al.*,^2^ (5.5% and 14.3%). Though we had a 3.5% incidence of significant epidural bleeding in the primary group, surprisingly we did not encounter this problem in the revision group. This could again be due to small numbers; the fact that during revision surgery we do not dissect through the epidural scar tissue and instead go laterally, and lastly that all the revision surgeries were performed by the senior author himself.

The current study has its inherent limitations: it is a retrospective study based on case records and imaging studies; numbers in the revision group are not large enough to be compared statistically with the primary surgery group; longer follow-up is required to give more insight into the results of revision surgery. The psychological profile and workers' compensation claims were not considered as their assessment was not practical in our setup.

## CONCLUSIONS

The present study reasserts that lumbar discectomy is a safe, simple and effective procedure with satisfactory outcome in 96.5% of primary disc surgeries and 78.6% of revision disc surgeries. The short-term recurrence rates with this procedure are also low (3.5%).
